# Sports engagement and age at first myocardial infarction in men under 55 years of age

**DOI:** 10.1371/journal.pone.0184035

**Published:** 2017-09-21

**Authors:** Christoph Janggen, Christoph Gräni, Jonas Brunner, Lukas D. Trachsel, Stephan Windecker, Prisca Eser, Lorenz Räber, Matthias Wilhelm

**Affiliations:** Department of Cardiology, Inselspital, Bern University Hospital, Bern, Switzerland; Indiana University School of Medicine, UNITED STATES

## Abstract

**Objective:**

Low levels of physical activity in childhood are associated with clustering of cardiovascular risk factors (CVRF) as predisposition for atherosclerosis. We assessed the association between sports engagement and age at first myocardial infarction (MI) in a cohort of men under 55 years of age.

**Methods:**

The Bern percutaneous coronary intervention Registry (NCT 02241291) was analyzed from March 2009 until January 2012. Male patients with first MI, age 18 to 54 years and body mass index ≤25kg/m^2^ were included. Patients were stratified into two groups based on their starting age with organized sports ≥1 h/week outside school (EARLY: <18, CONTROL: ≥18 years or never). We assessed age at time of first MI, CVRF, and volume of sports training.

**Results:**

Of 4,394 consecutive patients, 123 fulfilled the inclusion criteria (EARLY n = 81, CONTROL n = 42). Age at the time of first MI was 3 years younger in the EARLY compared to the CONTROL group (46.8±6.0 vs. 49.8±4.6 years, p = 0.006). Total lifetime training hours, and average yearly training hours, both, before and after age 18, were significantly greater in the EARLY group. Years of training <18 years were weakly inversely correlated with age at first MI (r^2^ = 0.075, p = 0.002). The proportion of sports-related MI was not different between EARLY and CONTROL (13.6% vs. 11.9%). Patients in the EARLY group had fewer CVRF (2 vs. 3; p = 0.001). Prevalence of smoking was equally high in both groups (63.0% and 64.3%).

**Conclusions:**

In our patients aged 54 and younger, the first MI occurred 3 years earlier in those who started regular sports activity before age 18, despite a more active lifestyle and favorable CVRF profile.

## Introduction

Atherosclerosis is a multifactorial, systemic, and chronic disease involving endothelial dysfunction and chronic inflammation [1]. Atherosclerosis begins in childhood and adolescence, and is aggravated by the presence of cardiovascular risk factors (CVRF) [2]. Regular physical activity (PA) is generally accepted as a cornerstone of cardiovascular disease prevention and current guidelines recommend at least 150 minutes per week of moderate exercise or 75 minutes per week of vigorous exercise or a combination thereof [3, 4]. PA induces laminar shear stress resulting in endothelial activation with the attendant reduction of oxidative stress, maintenance of the vessel wall integrity, and improved regulation of vascular tone and hemostasis [5]. Endurance exercise may also reduce circulating concentrations of inflammatory markers via release of anti-inflammatory cytokines provoked by skeletal muscle contraction, which inhibits tumor necrosis factor-alpha production in adipose tissue and macrophages [6–9]. Furthermore, exercise has a positive impact on CVRFs via modulation of insulin sensitivity, lipid profile, blood pressure, body weight and autonomic balance [9].

Low levels of PA in childhood are associated with a clustering of CVRF [10]. Ideal cardiovascular health in childhood has been shown to prevent cardiometabolic outcomes in adulthood [11]. Although childhood physical fitness and activity seems to reveal some inverse associations with CVRF in early adulthood, these effects may diminish markedly with increasing age [12]. The influence of early onset of regular exercise during childhood and adolescence on occurrence and timing of first time myocardial infarction (MI) later in life is currently unknown. In particular, in otherwise healthy persons with a genetic predisposition for CAD, it is not known whether engagement in sports is protective. The high likelihood of a genetic component in patients suffering MI at an age younger than 55 has been supported by a number of studies, such as the Health Family Tree Study, where individuals with a positive family history of CAD (FRS ≥0.5) represented only 14% of the general population but accounted for 72% of persons with early CAD (men before age 55 years) [13]. Furthermore, Roberts and colleagues suggested that more than one-half of CAD cases diagnosed before the age of 55 years are genetic [14].

We aimed to assess the association between sports engagement and age at first MI in a retrospective cohort study of normal-weight men presenting with a first MI at a young age of below 55 years, making the presence of a high-risk genotype probable [4, 15]. Our hypothesis was that men with an early start of sports activity during childhood or adolescence would pursue a more active lifestyle throughout their lifetime. Compared to their physically less active peers, this may positively affect CVRF profile and severity of coronary atherosclerosis and as a result postpone the time of a first MI. We assumed that patients with an active lifestyle would mostly have a normal body mass index (BMI), therefore we only included patients with BMI ≤ 25 kg/m^2^.

## Methods

### Study cohort

An established prospective registry of consecutive patients undergoing percutaneous coronary interventions (PCI) performed at the Bern University Hospital, Switzerland (CARDIOBASE Bern PCI Registry, NCT 02241291), was reviewed between March 2009 and January 2012. All consecutive male patients aged 18 to 54 years with a BMI ≤ 25kg/m^2^, and admitted with a diagnosis of acute, first MI were included. Definition of MI was based on established criteria, and MI was characterized as either Non-ST-segment elevation (NSTEMI), or ST-segment elevation MI (STEMI) [16]. To minimize gender bias we excluded female patients. Further exclusion criteria were history of known coronary artery disease (CAD) or previous MI. All patients provided written informed consent and the protocol was approved by the ethics committee of the Canton of Bern.

### Protocol

A self-designed questionnaire on sports history was conducted by telephone interview between February and April 2012. The main question was whether patients participated in organized sports (regular training) outside school physical education before age 18. Patients were stratified into two groups: Patients in the EARLY group performed at least 1 h of organized sports per week outside school for at least one year before age 18 and those in the CONTROL group did not engage in organized sports outside the compulsory sport at school. Sports level prior to the index event (average of last year prior to first MI) was defined as no sports, recreational sports (regular physical activity of ≥1 time/week), and competitive sports (participation in official athletic competitions, independently of the level of expertise [17]). In addition, physical activity was quantified as average yearly training hours, average yearly training hours before, as well as after age 18, and cumulative lifetime training hours.

CVRF, such as arterial hypertension, dyslipidemia, diabetes mellitus, family history regarding CAD and smoking were assessed based on established criteria [4]. A sedentary lifestyle was defined as absence of a minimum of 150 min of moderate-to-vigorous, or 75 min of vigorous physical activity per week or a combination thereof [3, 4]. Furthermore, body mass index, cardiac medication, left ventricular ejection fraction (LVEF), number of affected coronary arteries, numbers of implanted stents, diameter of stents, and laboratory test results (markers of myocardial injury, lipid profile, blood count) were recorded. Also, circumstances at time of MI (rest, light PA, recreational sport, competitive sport, emotional state) were recorded. Primary end-point was age at time of first MI.

### Statistical analyses

All statistical analyses were performed using SPSS Statistics for Windows, version 22 (IBM Corporation, Armonk, NY). Data are reported as mean±standard deviation (SD) or median (interquartile range, IQR) as appropriate. Continuous variables were analysed using the student’s T-test or Mann-Whitney U test, as appropriate. Categorical data were analysed with Fisher’s exact test. *P*-values of all outcomes were two-sided; an alpha of less than 0.05 was considered statistically significant. Spearman correlation coefficient was calculated to investigate the association between sports history and age of first MI. Linear regression models were performed for the dependent variable age at first MI and independent factors group allocation and smoking, and group allocation and sum of CVRF.

## Results

**Fig 1:**
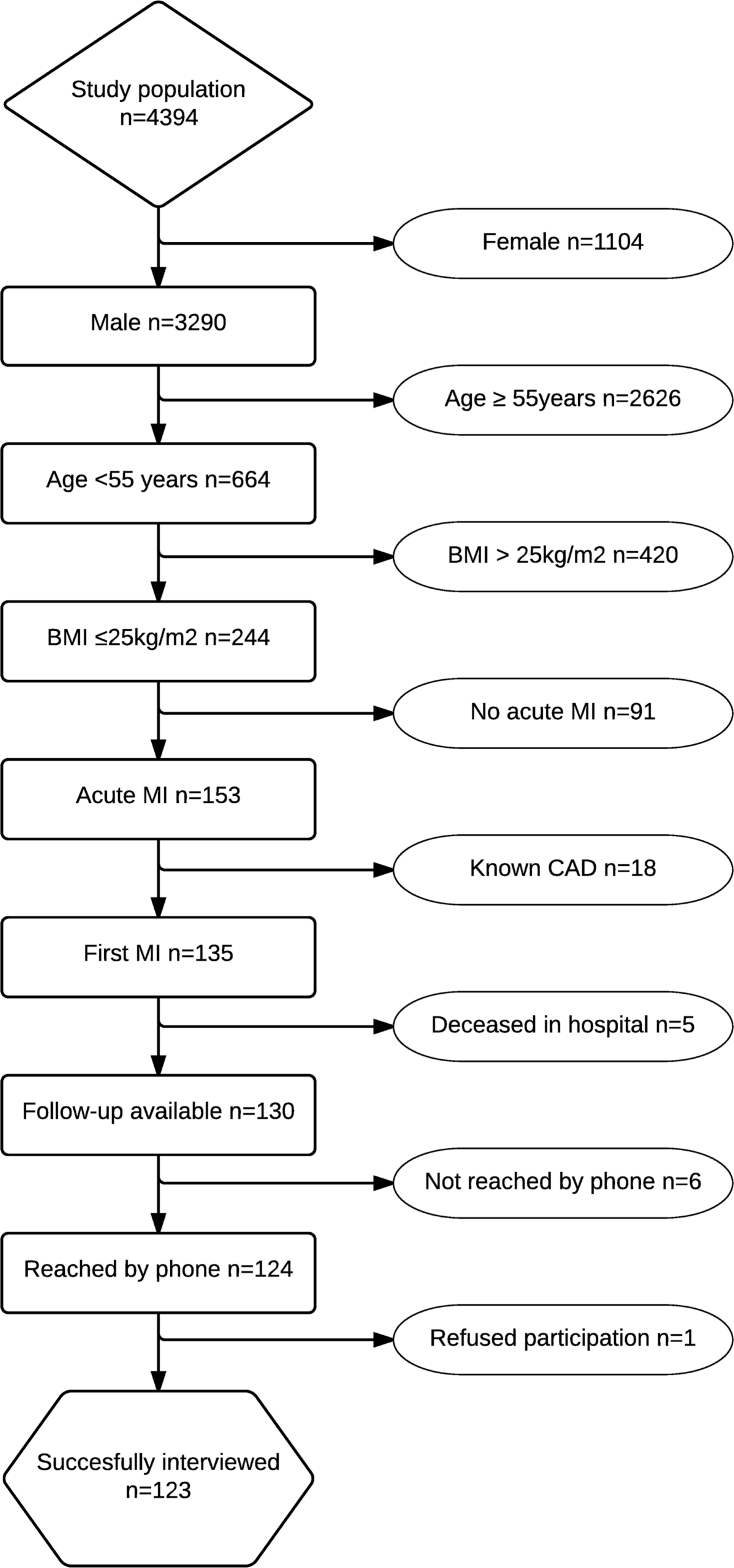
Study flow chart of the study population. BMI = body mass index; CAD = coronary artery disease; MI = myocardial infarction.

A total of 4,394 consecutive patients who underwent PCI at the Bern University Hospital between March 2009 and December 2012 were prospectively included in the registry. Of these, 135 (3.1%) patients fulfilled the inclusion criteria, and 123 patients were successfully interviewed as shown in the study flow chart (Fig 1).

A total of 81 (65.9%) patients engaged in regular sports activities of at least one hour per week outside school sports in their childhood and/or adolescence and were assigned to the EARLY group. The remaining 42 (34.1%) patients constituted the CONTROL group (Table 1). Cardiac medications prior to MI were not different between the groups. The most common sports disciplines amongst patients in the EARLY group was soccer (43.2%) followed by cycling (11.1%), ice hockey (8.6%), and running (6.2%), hand ball and athletics (3.7% each), and one or two patients (<2.5%) in each of the following sports disciplines: cross-country skiing, tennis, skiing, judo, table tennis, gymnastics, martial arts, mountaineering, land-hockey, swimming, wrestling, baseball. In the EARLY group, the proportion of patients engaged in recreational and competitive sports was higher, compared to CONTROL (p = 0.017), while fewer patients in the EARLY group did not participate in any sports (49% vs. 67%).

**Table 1:** Characteristics of patients in EARLY and CONTROL.

	EARLY (n = 81)	CONTROL (n = 42)	P value
Age at MI (years)	46.8±6.0	49.8±4.6	0.006
Body Mass Index (kg/m2)	23.4 [2.15]	23.9 [1.73]	0.048
*Sport level prior to MI*			
No Sports	40 (49.4%)	28 (66.7%)	
Recreational Sports	30 (37.0%)	14 (33.3%)	0.017
Competitive Sports	11 (13.6%)	0 (0%)	
*Physical activity*			
Total lifetime training hours	1600 [3225]	0 [500]	0.000
Average yearly training hours	38.2 [67.9]	0 [9.8]	0.000
Average yearly training hours before age 18	20 [24]	0 [0]	0.000
Average yearly training hours after age 18	1.5 [60.4]	0 [9.8]	0.025
*CVRF*			
Sum of CVRF	2 [1]	3 [2]	0.022
Hypertension	16 (19.8%)	14 (33.3%)	0.096
Hypercholesterolemia	24 (29.6%)	20 (47.6%)	0.048
Diabetes mellitus	5 (6.2%)	0 (0%)	0.165
Family history	29 (35.8%)	14 (33.3%)	0.785
Smoking (current)	51 (63.0%)	27 (64.3%)	0.885
Sedentary lifestyle*	56 (69.1%)	37 (88.1%)	0.026
*Medication prior to MI*			
Aspirin	11 (13.6%)	7 (16.7%)	0.789
Clopidogrel	5 (6.2%)	1 (2.4%)	0.663
Statin	11 (13.6%)	8 (19.0%)	0.440
ACE Inhibitor	3 (3.7%)	3 (7.1%)	0.410
AT II Antagonist	3 (3.7%)	2 (4.8%)	1.000
Beta-blocker	9 (11.1%)	6 (14.3%)	0.772

Parametric data is indicated as mean±SD, non-parametric data as median [interquartile range], and frequency data as number of cases (percentage of corresponding group). P-values are indicated for independent t-tests, Mann-Whitney test or Fisher’s exact test as appropriate.

* sedentary lifestyle was defined as less than 150 min physical activity per week on average. MI, myocardial infarction; CVRF, cardiovascular risk factors; ACE, Angiotensin Converting Enzyme; AT II, Angiotensin II

MI occurred most frequently at rest, followed by light physical activities, recreational sports, and competitive sports without differences between groups. The proportion of sports-related acute MI was not different between the groups (13.5% vs. 11.9%), (Table 2).

**Table 2:** Circumstances at the time of myocardial infarction (MI), type of MI, intervention, and laboratory results.

	EARLY (n = 81)	CONTROL (n = 42)	*P* value
*Circumstances at time of MI*			
Rest	36 (44.4%)	20 (47.6%)	
Light physical activity	14 (17.3%)	6 (14.3%)	
Recreational sports	10 (12.3%)	4 (9.5%)	0.519
Competitive sports	1 (1.2%)	1 (2.4%)	
Emotional state	3 (3.7%)	1 (2.4%)	
Others	0 (0%)	2 (4.8%)	
*Type of MI*			
STEMI	62 (76.5%)	24 (57.1%)	0.026
Multi-vessel disease	39 (48.1%)	29 (69.0%)	0.027
Ejection fraction (%)	50.0 [15.0]	50.0 [15.0]	0.075
*Intervention*			
Multi-vessel stenting	9 (11.1%)	10 (23.8%)	0.065
Total stent length (mm)	26.0 [22.3]	30.5 [22.75]	0.355
Mean stent diameter (mm)	3.1 [0.58]	3.0 [0.55]	0.006
*Laboratory results*			
Peak CK-MB (U/l)	118.6 [226.6]	71.15 [170.9]	0.040
Peak Troponin T (μg/l)	3.52 [4.74]	1.28 [4.25]	0.016
Creatinine (μmol/l)	72.0 [24.0]	77.0 [25.8]	0.328
Total cholesterol (mmol/l)	4.96 [1.40]	4.70 [1.98]	0.928
HDL cholesterol (mmol/l)	1.10 [0.52]	1.10 [0.37]	0.781
LDL cholesterol (mmol/l)	3.28 [1.32]	3.00 [1.75]	0.923
LDL ≥5 mmol/l	2 (2.5%)	4 (9.5%)	0.179
Triglycerides (mmol/l)	1.07 [0.75]	1.29 [1.27]	0.159
Leucocytes (G/l)	11.8 [4.65]	9.55 [4.57]	0.017

Frequencies in number of subjects (% of respective group) and p-values of Fisher’s exact tests are shown. Continuous data is shown as median [interquartile range], and p-values are derived from Mann-Whitney tests. MI, myocardial infarction; STEMI, ST-elevation MI; CK-MB, muscle-brain type creatine kinase; HDL, high-density lipoprotein; LDL, low-density lipoprotein

**Fig 2:**
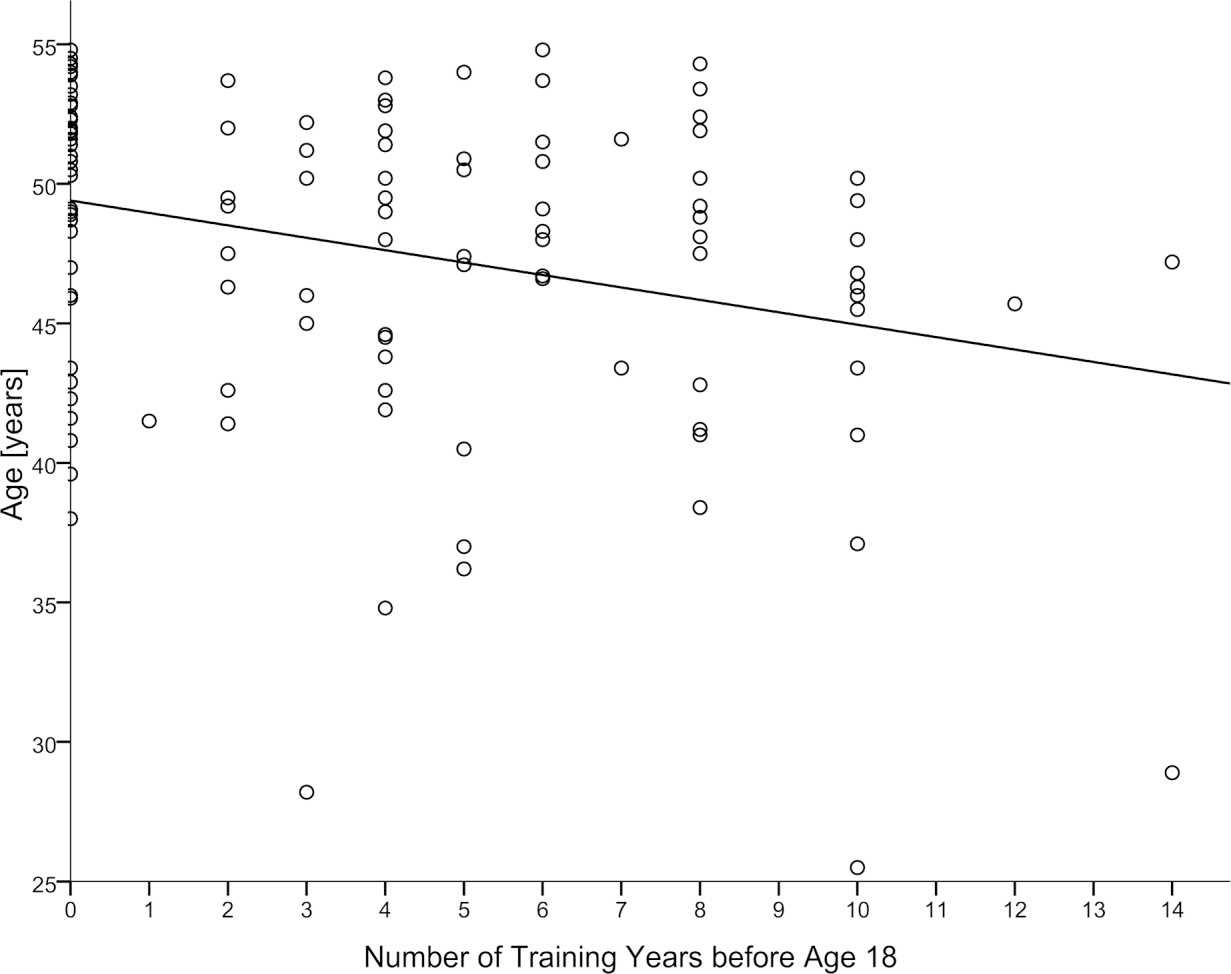
Relationship between age at first MI and years of training before age 18. Linear regression line is shown, the Spearman correlation coefficient is -0.277 (p = 0.002).

Age at manifestation of first MI was 3 years lower in the EARLY compared to the CONTROL group (p = 0.006). Total lifetime training hours, average yearly training hours, and average yearly training hours after age 18 were higher in the EARLY compared to CONTROL. Training years before age 18 was inversely correlated with age at MI, with a Spearman correlation coefficient of -0.277 (p = 0.002, Fig 2).

Patients in the EARLY group more frequently presented with STEMI and multi-vessel disease, as well as significantly higher peak creatine kinase (CK-MB), troponin T, and leukocytes levels. However, they tended to have less multi-vessel stenting. While total stent length was not different, mean stent diameter was significantly larger in EARLY.

**Fig 3:**
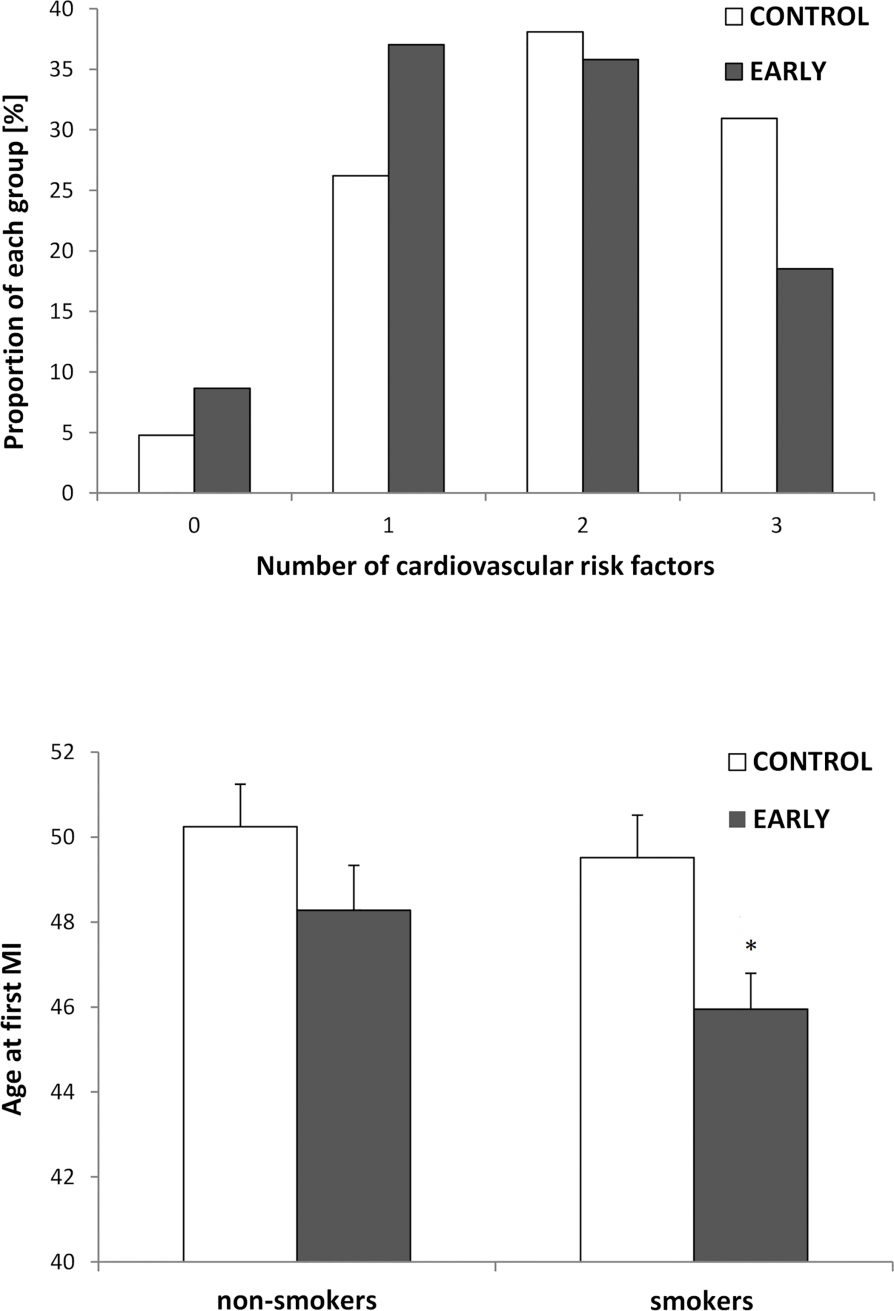
Cardiovascular risk factor (CVRF) profile of study population. Frequency of the sum of CVRFs of EARLY and CONTROL group (top panel) and mean age at first MI for smokers and non-smokers stratified according to EARLY and CONTROL (bottom panel). Error bars indicate standard errors. *EARLY significantly different from CONTROL with p = 0.004.

The average number of CVRF was lower in the EARLY compared with CONTROL group (2 vs. 3, p = 0.001), (Fig 3, top panel). The proportion of patients with a sedentary lifestyle at age of MI was lower in EARLY and fewer patients had hypercholesterolemia. Both groups were comparable for diabetes mellitus, family history of CAD, and prevalence of active smoking. Only 27% in the EARLY group and 19% in the CONTROL group were never smokers. In the EARLY group smokers had an earlier age of first MI compared to non-smokers (difference of 3.6 years, p = 0.008), (Fig 3, bottom panel).

In the linear regression models the interaction terms were non-significant and were therefore omitted from the model. In the model for age at MI with factors group allocation and smoking, only group allocation was significant (p = 0.009), with the model explaining 7% of total variance in age at MI. In the model with factors group allocation and sum of risk factors, again only group allocation was significant (p = 0.008), explaining 6% of total variance.

## Discussion

This is the first study assessing the association between sports engagement and the time point of the first acute manifestation of coronary atherosclerosis in a cohort of men under 55 years of age. Unexpectedly, we had to reject the initial hypothesis, that an early start of regular exercise would delay the occurrence of a first MI. The main finding of our study was that the age at first MI was significantly lower in patients with an early (<18 years) start of regular sports activity outside school physical education and that the phenotype of atherosclerosis was more aggressive. Among the group of EARLY versus CONTROL patients, a more active lifestyle, a lower sum of CVRF, a lower body mass index and a lower prevalence of hypercholesterolemia was observed. Despite the suggestion of a more favorable risk profile, the first acute manifestation of coronary artery disease was not only earlier but also more frequently caused by occlusive coronary thrombosis as indicated by the higher frequency of STEMI in the EARLY group. Consistent with the more frequent presentation of STEMI, patients in EARLY had evidence of more focal and proximally located CAD as indicated by less multi-vessel disease and a larger stent diameter. Also within the STEMI patients only, a significant 2.5 year difference remained between those that did and did not engage in sports activities before age 18.

Importantly, this cohort comprised a selected high-risk population with an early and acute manifestation of coronary atherosclerosis. Normal-weight men who suffer an acute MI at relatively young age are likely to have environmental and/or genetic risk factors [15, 18]. In the absence of genetic analyses in this study, we cannot provide further insights. The lower proportion of patients with known hypercholesterolemia (29.6% vs. 47.6%) or markedly elevated LDL cholesterol (≥ 5 mmol/l) (2.5% vs. 9.5%) in EARLY compared to CONTROL excludes familiar hypercholesterolemia as a causative factor for the early onset of MI in the EARLY group.

The prevalence of active smokers was high, both in EARLY and CONTROL, (63% and 64%), and it can be assumed that most of the smokers started smoking in adolescence [19]. The prevalence of former smoking was another 10% and 18% in EARLY and CONTROL, respectively, resulting into ever-smokers of 74% and 80%, respectively. This prevalence of baseline smoking was exactly twofold the Swiss smoking prevalence of men aged 45–54 in 2012 (Swiss Federal Statistical Office 2015)[20]. Smoking contributed significantly to a lower age at first MI in EARLY (Fig 3, bottom panel). Smoking reduces peripheral blood flow [21] and leads to endothelial dysfunction and chronic inflammation [22, 23]. Moreover, smoking has been associated with a reduced number of circulating endothelial progenitor cells (EPC) together with an impairment of EPC differentiation and functional activities [24]. EPCs are crucial for endothelial maintenance and repair [25]. PA increases peripheral blood flow and shear stress. If this applies to a vasculature altered by smoking [26] and in the absence of sufficient number of circulating EPCs, high shear stress may contribute to persistent endothelial injury and progression of cardiovascular disease [25]. Our observation appears to be in contrast to epidemiological data suggesting that physically active smokers have a higher life expectancy compared to sedentary smokers [27]. This data, however, was derived from a population-based study in persons aged ≥65 years, while our data stems from a carefully selected young patient group with few CVRFs, but high proportion of smoking and likely presence of a genetic component.

In athletes with high training volumes, some studies suggested that the risk for CAD may be either increased [28] or comparable to sedentary individuals [29, 30]. Furthermore, ball heading in soccer or other mild traumatic injuries from other sports disciplines may be associated with subclinical coronary atherosclerosis [31]. However, in our study, the association of sports history with early onset of MI has to be interpreted in the context of an overall low level of PA during adulthood in this population. Although this cohort was limited to normal-weight men in order to recruit a physically active group of MI patients, more than half of all EARLY subjects ceased their sports activity prior to the first MI, only 34.6% still participated in recreational sports, and 11.3% in competitive sports. Sports activity in a general Swiss population of comparable age was previously shown to be higher (73% recreational sports, 20% of those competitive sports) [32]. The yearly training hours across the entire lifespan as well as after the age of 18 tended to be higher in the EARLY group and thus, the results are debatable with regard to the timing of sports activities and occurrence of MI, i.e. whether it was the early start of sports activities or a greater lifetime training volume that showed the association with earlier onset of MI. The significant correlation of training years prior to age 18 with the age at first MI, the low yearly training hours in EARLY after the age of 18 as mentioned above, and the absence of a significant relationship between total lifetime training hours with age at MI are in support of the observed association between sports activity during childhood and adolescence and the earlier time point of MI onset. However, it may also be that years of sports engagement outside school physical education was the variable with the least recall bias of all of our variables quantifying sports engagement before and after age 18. Indeed, all variables of physical activity before and after age 18 differed significantly between groups.

The majority of acute MI occurred at rest or during light PA. Sports-related MIs were equally distributed between EARLY and CONTROL, excluding exercise-induced plaque rupture as a reason for the observed age difference at first MI in EARLY [33–35]. Interestingly, the white blood count was significantly higher in EARLY. Elevated leukocyte counts are a surrogate marker of inflammation and important mediators at the various stages of cardiovascular disease progression and complication [1, 36]. Elevated leukocytes and pro-inflammatory cytokines have been demonstrated after strenuous activities like marathon running [37]. Frequent marathon running has been associated with a high coronary artery calcium burden and subclinical myocardial damage, and bursts of inflammation during training and competition were possible explanations [38]. However, since lifetime training hours and average yearly training hours were relatively low in our population and only 11.3% of patients in EARLY had a history of sports competitions, a contribution of exercise-induced inflammation to CAD progression and the acute MI is a very unlikely explanation for the observed age difference at fist MI in our population. The most likely reason for a higher leukocyte count in EARLY was the higher proportion of STEMI in this group [36].

### Study limitations

The main limitation is the small sample size, which means that our results have generated a hypothesis that will need to be tested in a larger population. Further, we intended to include a physically active population by excluding individuals with BMI≤25 kg/m^2^, instead, we ended up with a relatively inactive population of mainly smokers. Sports history data may have been confounded by recall bias, especially the determination of lifetime training hours, but probably less so the number of training years before age 18. Since the database included only smoking status, start of smoking and pack years were not recorded. We included a selected high risk population of patients with an early manifestation of MI, and the results cannot be extend to the general population. To confirm our findings, a population based study would be required. As there is evidence that chronic inflammation and higher inflammatory markers measured in young adulthood is associated with a MI later in life [39], possible underlying infections or chronic rheumatic diseases, which were not recorded in our study, may have had an influence on our results. Further, we excluded females as MI in premenopausal women is extremely rare and would not have allowed us to reach a balanced population, therefore our results apply to men only. The phone questionnaire used was self-designed and not validated.

## Conclusions

In our sample of normal-weight men aged 54 and younger, a first MI occurred in average three years earlier in those patients who started regular sports activity before age 18, despite a more favorable CVRF profile. In the presence of genetic and/or environmental risk factors like smoking, sports activity at young age may lead to an earlier manifestation and more vulnerable phenotype of symptomatic atherosclerosis. Our observation challenges the concept that exercise is a medicine for everyone. Our results will need to be verified in further population based studies, which should assess the effect of exercise on the vasculature in the presence of environmental and genetic risk factors, not modifiable by exercise itself.

## Supporting information

S1 Table Minimal data set. (XLS)

S1 Text Sports questionnaire: This questionnaire was conducted as a phone interview. (DOC)
