# Supplementary material for: Sports engagement and age at first myocardial infarction in men under 55 years of age
Source: PLoS One. 2017 Sep 21;12(9):e0184035. doi: 10.1371/journal.pone.0184035 (PMC5608204; doi:10.1371/journal.pone.0184035)
Supplement: S1 Text — (DOC) [file pone.0184035.s002.doc]

**Sport Fragebogen**

**Einwilligung des Patienten**

Patient wurde aufgeklärt und willigt ein zur anonymen Publikation seiner Daten

∟ Nein (0) ∟Ja (1)

**Zeitpunkt MI**

∟ Kompetitiver Sport (1) ∟ Freizeitsport (2) ∟ Leichte körperliche Aktivität (3)

∟ In Ruhe (4) ∟ Während Nacht (5) ∟ Emotionaler Aufregung (6)

∟ Andere (7) ______________

**Sport vor erstem Myokardinfarkt**

∟ Nein (0) ∟Ja (1)

**Level vor MI**

∟ Kein Sport (0) ∟ Freizeitsport (1) ∟ Leistungssport (2) ∟ Profi (3)

**Sportart vor MI****

∟ Laufsport (1) ∟ Skilanglauf (2) ∟ Radfahren (3)

∟ Tennis (4) ∟ Fussball (5) ∟ Ice Hockey (6)

∟ Skifahren (7) ∟ Anderes (8) ______________

**Kumulatives Training Jahre, Stunden/Woche vor MI**

∟ Jahre (ab 18 LJ) _____________

∟ Stunden/Woche _____________

**Anzahl Wettkampfteilnahmen vor MI**

∟ Marathon (0) _____________ ∟ Rundlauf (1) _____________

∟ Skimarathon (2) _____________ ∟ Anderes (3) _____________

**Kumulatives Training, Jahre, Stunden/Woche vor 18. Lebensjahr**

∟ Jahre (vor 18 LJ, ausserhalb Schulsport) _____________

∟ Stunden/Woche _____________

∟ Sportart** _____________

**Sport nach erstem Myokardinfarkt**

∟ Nein (0) ∟Ja (1)

**Level nach MI**

∟ Kein Sport (0) ∟ Freizeitsport (1) ∟ Leistungssport (2) ∟ Profi (3)

**Sportart nach MI**

∟ Laufsport (1) ∟ Skilanglauf (2) ∟ Radfahren (3)

∟ Tennis (4) ∟ Fussball (5) ∟ Ice Hockey (6)

∟ Skifahren (7) ∟ Anderes (8) ______________

**Kumulatives Training, Jahre, Stunden/Woche nach MI**

∟ Jahre _____________ ∟ Pause _____________

∟ Stunden/Woche _____________

**Anzahl Wettkampfteilnahmen nach MI**

∟ Marathon (0) _____________ ∟ Rundlauf (1) _____________

∟ Skimarathon (2) _____________ ∟ Anderes (3) _____________

**Rehabilitation**

∟ Ja ∟ Nein (0)

∟ Stationär (1) ∟ Ambulant (2)

∟ Dauer_______________

CRF ausgefüllt am Datum (dd/mm/yyyy) ..…./….../ 2012

Von *Christoph Janggen* Signature………………....................

**Sports Questionnaire**

**Patient consent**

Patient was informed and consented to anonymous publication of his/her data

∟ no (0) ∟yes (1)

**Circumstances of MI**

∟ competitive sports (1) ∟ recreational sports (2) ∟ light physical activity (3)

∟ at rest (4) ∟ during night (5) ∟ emotional agitation (6)

∟ other (7) ______________

**Sports engagement before MI**

∟ no (0) ∟yes (1)

**Sports level before MI**

∟ no sports (0) ∟ recreational sports (1) ∟ competitive sports (2) ∟ professional (3)

**Sports discipline before MI****

∟ running (1) ∟ cross-country skiing (2) ∟ cycling (3)

∟ tennis (4) ∟ soccer (5) ∟ Ice Hockey (6)

∟ skiing (7) ∟ other (8) ______________

**Cumulative training years, hours/week before MI**

∟ number of years (age> 18 yrs) _____________

∟ hours/week _____________

**Number of competitions before MI**

∟ marathon (0) _____________ ∟ running event (1) _____________

∟ skimarathon (2) _____________ ∟ other (3) _____________

**Cumulative training, years, hours/week before age 18**

∟ number of years (before age 18, additional to school physical education) _____________

∟ hours/week _____________

∟ sports discipline** _____________

**Sports engagement after MI**

∟ no (0) ∟yes (1)

**Sports level after MI**

∟ no sports (0) ∟ recreational sports (1) ∟ competitive sports (2) ∟ professional (3)

**Sports discipline after MI**

∟ running (1) ∟ cross-country skiing (2) ∟ cycling (3)

∟ tennis (4) ∟ soccer (5) ∟ Ice Hockey (6)

∟ skiing (7) ∟ other (8) ______________

**Cumulative training years, hours/week after MI**

∟ years _____________ ∟ intermission _____________

∟ hours/week _____________

**Number of competitions after MI**

∟ marathon (0) _____________ ∟ running event (1) _____________

∟ skimarathon (2) _____________ ∟ other (3) _____________

**Rehabilitation**

∟ yes ∟ no (0)

∟ stationary (1) ∟ ambulatory (2)

∟ duration_______________

CRF completed on date (dd/mm/yyyy) ..…./….../ 2012

By *Christoph Janggen* Signature………………....................
